# Cytomegalovirus-specific CD154-expressing T Cells are Present Before Transplantation in Cytomegalovirus-seronegative Recipients and Predict Early Cytomegalovirus DNAemia

**DOI:** 10.1097/TXD.0000000000001923

**Published:** 2026-02-23

**Authors:** Lisa Remaley, Kyle Soltys, Tamara Fazzolare, Mylarappa Ningappa, Michael Green, Marian Michaels, Morgan Paul, Brianna Spishock, Dawn Wilkerson, Vikram Raghu, Brandon W Higgs, George Mazariegos, Ajay Khanna, Armando Ganoza, Geoffrey Bond, Chethan Ashokkumar, Rakesh Sindhi

**Affiliations:** 1 Department of Surgery, School of Medicine, Hillman Center for Pediatric Transplantation, UPMC Children’s Hospital of Pittsburgh, Pittsburgh, PA.; 2 Plexision, Pittsburgh, PA.

## Abstract

**Background.:**

Cytomegalovirus (CMV) antiviral drugs can cause side effects and promote the emergence of drug-resistance after transplantation. T cell–mediated immunity to CMV (CMI) can be used to limit and optimize the duration of antiviral therapy. However, interferon-gamma release assays show varying rates of CMI in CMV-seronegative recipients (R^–^), who are at the highest risk of CMV infection with CMV-seropositive donors (D^+^). Here, we characterize CMI with CMV-specific T cells that express CD154 in R^–^ liver transplant and intestine transplant recipients.

**Methods.:**

Pretransplant blood leukocyte samples from 42 R^–^ recipients were stimulated with a 15-mer overlapping peptide mixture representing the pp65 CMV antigen. CMI was measured by flow cytometry with CMV-specific CD154^+^ T-cell frequencies.

**Results.:**

Recipients, median age (range) 3.3 (0.4–36.5 y), included 39 liver transplant and 3 intestine transplant. CMV serostatus was D^+^/R^–^ in 21, and D^–^/R^–^ in 21. Protective CMI levels of >1.7% previously established in 142 liver or intestine recipients were observed in 25 of 42 total R^–^ recipients (60%), including 11 of 21 D^+^/R^–^ (52%) and 14 of 21 D^–^/R^–^ (67%) participants. CMV DNAemia was observed in the first 60 d after transplantation in 10 of 42 (24%) R^–^ recipients, including 8 of 17 with CMI <1.7% and 2 of 25 with CMI >1.7%. Adjusted for D^+^/R^–^, CMI<1.7% was independently associated with a higher risk of DNAemia (hazard ratio 6.04 [1.2-29.4], *P* = 0.026).

**Conclusions.:**

CMV-specific T cells that express CD154 demonstrate protective levels of CMI in half of all seronegative recipients and can be used to optimize antiviral prophylaxis across all risk categories.

## INTRODUCTION

Cytomegalovirus (CMV) risk stratification is currently based on donor-recipient serologic mismatch and needs improvement to optimize antiviral drug use after transplantation.^1^ CMV is 1 of the most common posttransplant infections and is associated with higher rates of rejection, graft loss, and death.^2^ Prophylactic antiviral drugs are used to prevent CMV infection or reactivation after transplantation with CMV-seropositive donors and/or recipients. These drugs can cause marrow suppression in a third of all recipients and promote the emergence of drug-resistant CMV with prolonged use.^3,4^ These risks are highest among seronegative recipients of organs from seropositive donors, who may receive 3–12 mo of antiviral drug prophylaxis, and in whom drug-resistant CMV can affect 3%–16%.^5-7^ CMV infection is associated with loss of T cell–mediated immunity (CMI) to CMV, and the presence of protective levels of CMI has been successfully used as an endpoint for early termination of antiviral drugs in a recent adult transplant study.^8^ A positive interferon-gamma (IFN-γ) release assay (IGR) result, defined as ≥ 0.2 IU/mL, implied protective levels of CMI in this study and was observed in 35 of 60 R^+^ (58%) and 1 of 39 (2.6%) D^+^/R^–^ recipients (2.6%). This limitation of IGR assays in seronegative recipients needs further study, given the significant and progressive increase in the incidence of D^+^/R^–^ transplants among >470 000 recipients in the Scientific Registry of Transplant Recipients (2000–2020).

Intracellular staining after cell permeabilization and flow cytometry analysis has shown that IFN-γ-expressing CMV-specific T cells coexpress CD154 (CD40LG).^9^ The expression of CD154 on T cells can also be detected with a less cytotoxic nonpermeabilizing method, potentially increasing the numbers of detectable CMV-specific T cells. We have used CD154 in lieu of IFN-γ to identify CMV-specific T cells and characterized CMI in 40 healthy adults and 142 recipients of liver or intestinal allografts, of whom a fourth were young adults older than 18 y.^9^ In this study, CMV-specific CD154^+^ T-cell frequencies of ≥1.7% predicted freedom from CMV DNAemia with a negative predictive value of 93%. Although average CMV-specific T-cell frequencies were lower in samples from seronegative recipients compared with seropositive recipients, frequencies of ≥1.7% were present in half of these seronegative recipients, creating opportunities for future antiviral drug optimization strategies in D^+^/R^–^ recipients.

Here, we evaluate CMV-specific CD154^+^ T-cell frequencies and their association with early CMV DNAemia in an expanded cohort of pretransplant leukocyte samples from 42 seronegative liver transplant (LT) and intestine transplant (IT) recipients, including 20 reported previously.

## MATERIALS AND METHODS

All participants were enrolled in the study through University of Pittsburgh IRB-approved protocols No. 0405628 and No. 1430156 with written consent. Pretransplant blood leukocyte samples from 42 R^–^ recipients were stimulated with a 15-mer overlapping peptide mixture representing the pp65 CMV antigen. CMV-specific CMI was measured by CD154^+^ T-cell frequencies using flow cytometry. The presence and timing of CMV DNAemia during the first 60 d after transplantation were recorded. Two weeks of prophylactic intravenous ganciclovir were administered to all D^+^/R^–^ and R^+^ recipients, and D^–^/R^–^ IT recipients. DNAemia was measured with PCR. Our center uses a surveillance-after-prophylaxis approach, with antiviral drug prophylaxis followed by PCR surveillance of CMV viral load. Two weeks of IV ganciclovir or oral valcyte is given to all recipients, except D^–^/R^–^ liver recipients who do not receive any prophylaxis, and D^+/^R^–^ recipients of intestine-containing allografts who receive 3 mo of ganciclovir/valcyte prophylaxis supplemented with cytogam. Statistical analyses were conducted using SPSS version 31 (IBM Corporation, NY).

## RESULTS

### Participants

Forty-two recipients, median age (range) 3.3 (0.4–36.5 y), included 8 participants (19%) 18 y or older. Recipients included 20 male, 22 female, 35 White, 7 non-White individuals who received 38 liver, 1 liver/heart, and 3 multivisceral grafts. Indications for LT were biliary aresia (9), urea cycle defects (11), Criggler-Najjar syndrome (1), metabolic disease (2), maple syrup urine disease (7), progressive familial intrahepatic cholestasis (2), primary sclerosing cholangitis (1), methymalonic acidemia (1), propionic acidemia (1), hepatoblastoma (4), hepatic fibrosis (1), gastroschisis (1), and pseudo-obstruction (2). CMV serostatus was D^+^/R^–^ in 21, and D^–^/R^–^ in 21. All D^+^R^–^ received prophylactic antiviral drug for a median of 14 d (6–24 d) from transplant.

**FIGURE 1.**
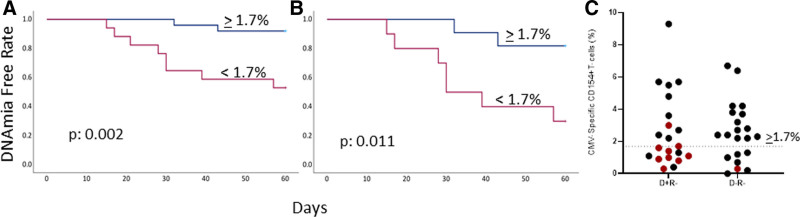
DNAemia-free rate within 60 d after transplantation in 42 R^–^ (A) and 21 D^+^/R^–^ recipients (B) with CMI ≥1.7% and <1.7%. C, Dot plot shows distribution of CMI ≥1.7% and <1.7% in 21 D^+^/R^–^ and 21 D^–^/R^–^ recipients. Dotted line identifies CMI level of ≥1.7%. Black dots represent no DNAemia and red dots represent DNAemia. D, donor; CMI, T cell–mediated immunity; R, recipient.

CMV DNAemia was observed in the first 60 d after transplantation in 10 of 42 (24%) R^–^ recipients, with a median interval of 29 d (15–57). DNAemia ranged from 650 IU/mL to 16 985 IU/mL with a median of 4285 IU/mL. Nine of 10 cases occurred in the D^+^/R^–^ group (9/21; 43%), and 1 of 21 occurred in the D^–^R^–^ group (1/21; 5%). CMV-specific CD154^+^ T cells were detected in 41 of 42 R^–^ recipients with median frequencies 2.3% (range 0.2% to 9.3%; Table 1). Protective CMI levels of ≥1.7% were observed in 25 of 42 total R^–^ recipients (60%), including 11 of 21 D^+^/R^–^ (52%) and 14 of 21 D^–^/R^–^ (67%) participants (Figure 1). R^–^ recipients with low CMI <1.7% experienced a higher incidence rate of developing DNAemia (*P* = 0.002, Kaplan-Meier and hazard ratio of 7.75 [95% confidence interval, 1.6-36.6], *P* = 0.01). Adjusting for D^+^/R^–^ recipients, CMI <1.7% was independently associated with increased risk of DNAemia (*P* = 0.011 Kaplan-Meier and hazard ratio of 6.04 [95% confidence interval, 1.2-29.4], *P* = 0.026). The only DNAemia episode among D^–^/R^–^ recipients occurred 21 d after transplantation in 1 of 7 recipients with low CMI.

**TABLE 1.** CMI distribution and DNAemia in test cohort and subcohorts

Serostatus	CMI ≥1.7%	N	CMI %, median (range)	No DNAmeia	DNAemia	*P*, K-M	Days to DNAemia, median (range)
All	No	17	1.0 (0–1.6)	9	8 (47%)	0.002	29 (15–57)
	Yes	25	3.2 (1.7–9.3)	23	2 (8%)		38 (32–43)
D^**+**^**/**R^–^	No	10	1.1 (0.3–1.6)	3	7 (70%)	0.011	30 (15–57)
	Yes	11	3.6 (1.7–9.3)	9	2 (18%)		38 (32–43)
D^–^/ R^–^	No	7	0.7 (0–1.3)	6	1 (14.2%)	NS	21
	Yes	14	3.0 (2.2–6.7)	14	0 (0%)		NA

D, donor; CMI, T cell–mediated immunity; CMV, cytomegalovirus; –, CMV IgG seronegative; +, CMV IgG seropositive; K-M, Kaplan-Meier; NS, not significant; R, recipient.

## DISCUSSION

Our study expands on our previous report by showing that CD154-expressing T cells can detect CMI to CMV before LT or IT in roughly half of CMV-seronegative (R^–^) recipients (Table 1, Figure 1). These observations are clinically relevant. Pretransplant frequencies of ≥1.7%, which predicted freedom from DNAemia in our previous report, are associated with reduced incidence and delayed onset of CMV DNAemia within the first 60 d after transplantation in this study. Noteworthy features of CMI measured with CD154-expressing CMV-specific T cells also include a large dynamic range of 0% to 9.3% T cells, and rapid kinetics of CD154 expression, which permit test reporting within 6 h. To test whether pretransplant CMI can predict DNAemia, we have restricted our observations to the 60-d period immediately after transplantation to minimize the possible effects of longer posttransplant immunosuppression on CMI and the development of CMV DNAemia. The predictive utility beyond 60 d after measurement of CMI is not known.

Our findings mirror those from several previous reports (Table 2), which show that CMI to CMV is present in 28%–57% of seronegative recipients and is associated with a lower incidence of DNAemia.^8-16^ Although CMI was measured with IGR assays or intracellular IFN-γ in most of these studies, at least 1 study used the alternative marker CD137 and showed that CD137-expressing CMV-specific T cells were present in 57% of recipients and also predicted freedom from DNAemia. However, only 9% of CD137^+^CD8^+^ T cells produced IFN-γ in CMV-seronegative individuals, compared with 36% CMV-seropositive individuals.^16^ Thus, IFN-γ-expressing CMV-specific T cells may be less suited to capture CMI among seronegative recipients. This limitation is compounded in IGR assays, where secreted IFN-γ likely originates from multiple cells and may underrepresent CMV-specific T cells. Protective IFN-γ-independent CMI has also been observed among individuals heavily exposed to Mycobacterium tuberculosis who failed to develop latent infection, but in whom both the tuberculin skin test and antigen-specific IFN-γ^+^ T cells were absent.^17^ This “resistant” state was best explained by the presence of mycobacterial antigen-specific CD154-expressing T cells in these heavily exposed individuals.

**TABLE 2.** Summary of published reports describing CMI to CMV in seronegative recipients of kidney (K), liver (Li), heart (H), lung (Lu), pancreas (P), and intestine (I) allografts

Author	Reference	Time from Tx	N	D/R status	Protective CMI	Subthreshold CMI	Method	CMV incidence	Organ type
Solera^8^	PMID: 39160648	Post	39	D^+^/R^–^	1 of 39 (2.6%)	38 of 39 (97.4%)	Qf-CMV	0 of 1 (0%) vs 15 of 39 (38%)	K, Li, H, P
Kumar^10^	PMID: 19422346	Pre & Post	35	D^+^/R^–^	10 of 35 (29%)	25 of 35 (71%)	Qf-CMV	1 of 10 (10%) vs 10 of 25 (40%)	Li, Lu, K, P
Andreani^11^	PMID: 31889538	Post	12	D^+^/R^–^	6 of 12 (50%)	6 of 12 (50%)	Qf-CMV	1 of 6 (17%) vs 4 of 6 (67%)	K
Veit^12^	PMID: 33749938	Post	50	D^+^/R^–^	14 of 50 (28%)	36 of 50 (72%)	ELISPOT	3 of 14 (21%) vs 19 of 36 (53%)	Lu
Schachtner^13^	PMID: 28594749	Pre-	67	D^+^/R^–^	19 of 67 (28%)	48 of 67 (72%)	ELISPOT	2 of 19 (11%) vs 17 of 48 (35%)	K
Lúcia^14^	PMID: 25048845	Pre-	43	R^–^	15 of 43 (35%)	28 of 43 (65%)	ELISPOT	1 of 15 (6%) vs 10 of 28 (36%)	K
Bestard^15^	PMID: 23711167	Pre-	28	D^+^/R^–^	12 of 28 (43%)	16 of 28 (57%)	ELISPOT	1 of 12 (8%) vs 8 of 16 (50%)	K
Litjens^16^	PMID: 28955345	Pre-	28	R^–^	16 of 28 (57%)	12 of 28 (43%)	FACS	higher in subthreshold CMI (*P* = 0.043)	K
Ashokkumar^9^	PMID: 31657119	Post	12	R^–^	6 of 12 (50%)	6 of 12 (50%)	FACS	1 of 6 (17%) vs 5 of 6 (83%)	Li, I
Remaley	Current study	Pre-	34	R^–^	20 of 34 (59%)	14 of 34 (41%)	FACS	1 of 20 (5%) vs 5 of 14 (36%)	Li, I

CMI, T cell–mediated immunity; Qf-CMV, Quantiferon-Cytomegalovirus; ELISPOT, enzyme-linked immunosorbent assay; FACS, fluorescence-activated cell sorting (also termed flow cytometry); Tx, transplant.

The low to variable incidence of CMI with IGR assays in R^–^ recipients, ranging from 2.6% to >50%, can lead to the inference that humoral and cellular immunity are highly correlated and develop together. The published evidence and the current study refute this inference (Table 2) and evoke several questions worth consideration: Can serologic status alone constitute evidence of prior exposure and risk of reactivation, independent of protective CMI in the recipient? If reactivation risk is a composite of prior exposure and protective CMI, should it be determined with a combined assessment of serologic status and CMI to further optimize the duration of antiviral prophylaxis? If IGR assays underestimate protective CMI in seronegative patients, should alternative CMI assays, such as CD154-expressing CMV-specific T cells, be used to assess risk across all CMV-risk categories? The impetus for change lies in the increasing recognition and development of multidrug-resistant CMV strains, the associated morbidity of alternative antiviral drugs, reactive reductions in immunosuppression that can increase the risk of rejection, and the resulting increased hospital stays and healthcare costs. Up to 30% of samples tested for CMV resistance in a recent study showed resistant strains, of which 8% were resistant to ≥2 drugs.^18^

### Limitation

We acknowledge the relatively small sample size and the potential risk of overfitting, given that 20 of the 42 participants in this study were previously included in the development of the predictive threshold using a training set of 46 participants. The predictive threshold was established with forward and backward logistic regression, incorporating additional covariates. The predictive threshold derived from the training set was independently validated in an additional cohort of 99 participants within the same prior study. Importantly, in the current analysis, the previously established threshold was applied without reoptimization or adjustment, and it demonstrated acceptable predictive performance in the expanded cohort of 42 pretransplant patients. This supports the applicability of the threshold despite the limited sample size. The association of CMV-specific T cells with freedom from DNAemia, as described in studies cited here, including the current study, does not imply causality; this has been established in part by the adoptive transfer of CMV-specific T cells to treat refractory disease.^19^

## CONCLUSIONS

Serial assessments of CMI can provide dynamic risk assessment in the course of predominantly T cell–directed immunosuppression. In turn, this approach can better inform antiviral drug use to limit side effects and drug-resistant CMV, at the very least in high-risk populations. These populations include pediatric recipients, among whom the proportion of R^–^ recipients is higher than in adults, for example, 63% versus 37%, respectively, among 62 945 total LT recipients (V. Raghu, personal communication, Scientific Registry of Transplant Recipients registry 2014–2022). Other vulnerable populations include intestine, multivisceral, and lung transplant patients, and those who have received lymphocyte-depleting immunosuppression. Surveillance with CMV-specific CD154-expressing T cells can occur before and after completing prophylaxis and preemptive treatment, or as needed if antiviral drug toxicity complicates antiviral drug use. The presence of protective CMI or persistently low CMI levels can, respectively, support decisions for early termination or continuation of antiviral drugs, provided other clinical features are also aligned.

## References

[1] Imlay H Wagener MM Vutien P. Increasing proportion of high-risk cytomegalovirus donor-positive/recipient-negative serostatus in solid organ transplant recipients. Transplantation. 2023;107:988–993. 36173456 10.1097/TP.0000000000004352 PMC10050221

[2] Hakimi Z Aballéa S Ferchichi S. Burden of cytomegalovirus disease in solid organ transplant recipients: a national matched cohort study in an inpatient setting. Transpl Infect Dis. 2017;19:e12732–e12735. 10.1111/tid.12732 28599091

[3] Abu-Omar A Mihm J Bronder S. CMV management of patients with leukopenia after CMV high-risk kidney transplantation. Transpl Immunol. 2025;89:102188. 39892765 10.1016/j.trim.2025.102188

[4] Fishman JA. Prophylaxis, preemption and drug resistance in CMV infection: too little, too much or just right? Am J Transplant. 2012;12:13–14. 21951581 10.1111/j.1600-6143.2011.03764.x

[5] Myhre HA Haug Dorenberg D Kristiansen KI. Incidence and outcomes of ganciclovir-resistant cytomegalovirus infections in 1244 kidney transplant recipients. Transplantation. 2011;92:217–223. 21685829 10.1097/TP.0b013e31821fad25

[6] Couzi L Helou S Bachelet T. High incidence of anticytomegalovirus drug resistance among D+R- kidney transplant recipients receiving preemptive therapy. Am J Transplant. 2012;12:202–209. 21967659 10.1111/j.1600-6143.2011.03766.x

[7] Kotton CN Kumar D Manuel O; on behalf of The Transplantation Society International CMV Consensus Group. The fourth international consensus guidelines on the management of cytomegalovirus in solid organ transplantation. Transplantation. 2025;109:1066–1110. 40200403 10.1097/TP.0000000000005374 PMC12180710

[8] Solera JT Ferreira VH Cervera C. Cell-mediated immunity to guide primary prophylaxis for CMV infection in organ transplant recipients: a multicenter single-arm prospective study. Transplantation. 2025;109:527–535. 39160648 10.1097/TP.0000000000005173

[9] Ashokkumar C Green M Soltys K. CD154-expressing CMV-specific T cells associate with freedom from DNAemia and may be protective in seronegative recipients after liver or intestine transplantation. Pediatr Transplant. 2020;24:e13601. 31657119 10.1111/petr.13601

[10] Kumar D Chernenko S Moussa G. Cell-mediated immunity to predict cytomegalovirus disease in high-risk solid organ transplant recipients. Am J Transplant. 2009;9:1214–1222. 19422346 10.1111/j.1600-6143.2009.02618.x

[11] Andreani M Albano L Benzaken S. Monitoring of CMV-specific cell-mediated immunity in kidney transplant recipients with a high risk of CMV disease (D+/R-): a case series. Transplant Proc. 2020;52:204–211. 31889538 10.1016/j.transproceed.2019.11.002

[12] Veit T Pan M Munker D. Association of CMV-specific T-cell immunity and risk of CMV infection in lung transplant recipients. Clin Transplant. 2021;35:e14294. 33749938 10.1111/ctr.14294

[13] Schachtner T Stein M Reinke P. CMV-specific T cell monitoring offers superior risk stratification of CMV-seronegative kidney transplant recipients of a CMV-seropositive donor. Transplantation. 2017;101:e315–e325. 28594749 10.1097/TP.0000000000001825

[14] Lúcia M Crespo E Melilli E. Preformed frequencies of cytomegalovirus (CMV)-specific memory T and B cells identify protected CMV-sensitized individuals among seronegative kidney transplant recipients. Clin Infect Dis. 2014;59:1537–1545. 25048845 10.1093/cid/ciu589 PMC4650765

[15] Bestard O Lucia M Crespo E. Pretransplant immediately early-1-specific T cell responses provide protection for CMV infection after kidney transplantation. Am J Transplant. 2013;13:1793–1805. 23711167 10.1111/ajt.12256

[16] Litjens NHR Huang L Dedeoglu B. Protective cytomegalovirus (CMV)-specific T-cell immunity is frequent in kidney transplant patients without serum anti-CMV antibodies. Front Immunol. 2017;8:1137. 28955345 10.3389/fimmu.2017.01137 PMC5600906

[17] Lu LL Smith MT Yu KKQ. IFN-γ-independent immune markers of Mycobacterium tuberculosis exposure. Nat Med. 2019;25:977–987. Erratum in: Nat Med. 2019 Jul;25(7):1175. doi: 10.1038/s41591-019-0519-y. PMID: 31110348; PMCID: PMC6559862. 31110348 10.1038/s41591-019-0441-3 PMC6559862

[18] Kleiboeker SB. Prevalence of cytomegalovirus antiviral drug resistance in transplant recipients. Antiviral Res. 2023;215:105623. 37150409 10.1016/j.antiviral.2023.105623

[19] Sun G Tu J Tang B. Adoptive transfer of third-party donor CMV-specific T cells for refractory cytomegalovirus infection following umbilical cord blood transplantation. Br J Haematol. 2025;207:2072–2079. 40931393 10.1111/bjh.70136

